# Impact of the COVID-19 pandemic on lung cancer diagnosis in northern Poland–addressing the COVID-19 debt

**DOI:** 10.1371/journal.pone.0316261

**Published:** 2024-12-27

**Authors:** Anna Romaszko-Wojtowicz, Anna Doboszyńska, Anna Piechnik, Krzysztof Kuziemski, Dariusz Szplit, Szczepan Cofta, Katarzyna Glińska-Lewczuk

**Affiliations:** 1 Department of Pulmonology, School of Public Health, Collegium Medicum, University of Warmia and Mazury in Olsztyn, Olsztyn, Poland; 2 Department of Lung Diseases, Neoplasms and Tuberculosis Faculty of Medicine, Collegium Medicum in Bydgoszcz, Nicolaus Copernicus University, Bydgoszcz, Poland; 3 Department of Pulmonology and Allergology, Faculty of Medicine, Medical University of Gdansk, Gdansk, Poland; 4 Health Technology Assessment and Implementation Team, University Clinical Center in Gdansk, Gdansk, Poland; 5 Department of Pulmonology, Allergology and Respiratory Oncology, Poznan University of Medical Sciences, Poznan, Poland; 6 Department of Water Resources, Climatology and Environmental Management, University of Warmia and Mazury in Olsztyn, Olsztyn, Poland; Mayo Clinic Rochester, UNITED STATES OF AMERICA

## Abstract

**Introduction:**

Lung cancer, one of the leading causes of death due to neoplasms, requires prompt diagnosis and immediate treatment. The COVID-19 pandemic affected healthcare systems worldwide, having adverse effects on all aspects, particularly on the fate of patients with suspected neoplastic diseases. Limited access to healthcare, disruptions in regular operations (reassigning roles to some wards), postponed hospital admissions, prolonged diagnostic processes, and other factors have collectively led to the phenomenon known as COVID-19 debt.

**Material and methods:**

A retrospective analysis covered statistical data concerning the diagnosis of lung cancer obtained from three centres in northern Poland (Olsztyn, Bydgoszcz, Gdansk) and concerning years 2016 to 2022. Relative risks (RR) with 95% confidence intervals (CI) for cancer event were calculated. Before the pandemic, these centers prioritized the diagnosis of suspected lung cancer cases, which was subsequently disrupted during the pandemic due to various factors.

**Results:**

The COVID-19 pandemic led to a decrease in diagnosed lung cancer cases, especially in hospitals repurposed for COVID-19 care. A statistically significant trend in lung cancer incidence per 100,000 inhabitants was observed specifically in healthcare centers that maintained normal operations without disruption.

**Conclusion:**

The concept of the COVID debt helps explain changes in lung cancer diagnosis during and post-pandemic, highlighting the need for increased public awareness and intensified diagnostic efforts to facilitate earlier disease detection.

## Introduction

The COVID-19 pandemic has profoundly affected healthcare systems worldwide, disrupting various aspects of medical care. Limited access to physicians, challenges in making timely diagnoses, and patients’ fear of infection have all had detrimental effects on public health, particularly affecting individuals suspected of having neoplastic diseases. Lung cancer stands as the leading cause of cancer-related mortality globally [1]. Early and timely diagnosis, coupled with immediate access to treatment, play pivotal roles in determining patient prognosis [2]. In 2020, Poland reported 204,575 new cancer cases, with lung cancer comprising the largest proportion at 14.4% (25,509 cases) [3]. However, there was a notable decrease in cancer detection in 2021, with 84,275 total cases diagnosed, and lung cancer accounting for 14.6% of these cases, indicating a substantial decline in detections. Current data on the ongoing impact of the COVID-19 pandemic on lung cancer diagnosis remain limited. Existing statistics from various regions worldwide mainly reflect the pre-pandemic period, with little information on the post-pandemic years. Reyes et al., in a retrospective Spanish study, reported a 38% decrease in new lung cancer diagnoses during the pandemic compared to pre-pandemic levels [4]. Similarly, northeastern Brazil saw a 42.7% reduction in new lung cancer diagnoses [5]. Mangone et al., through a comparative analysis of oncological data from northern Italy in 2019 and 2020, noted a 22% decrease in cancer diagnoses, including lung cancer, during the COVID-19 pandemic [6]. Delayed diagnosis in patients with chronic and progressive diseases worsens the prognosis for the individual patient, while at a population level, it creates a certain form of debt, which we have termed "COVID-19 debt" for the purposes of our publication.

This kind of backlog likely extends to many other chronic diseases, the treatment and prognosis of which rely on earlier detection. Reflecting on this issue shortly after the end of the COVID-19 pandemic underscores the urgent need for actions to address backlogs in diagnostics and treatment. Slotman and colleagues, in a survey conducted in European radiotherapy centers, observed a decrease in the number of screening tests and an increase in the number of patients presenting with advanced disease in one or more instances [7]. However, on the other hand, Van Hanren et al. noted a significant increase in the number of suspicious lung nodules after resuming screening programs in their facilities, from 8.0% before the pandemic to 29.0% during the pandemic [8].

However, research investigating the long-term impact of the COVID-19 pandemic on lung cancer detection remains insufficient. This study aims to evaluate the influence of the COVID-19 pandemic on the diagnostic process and detection of lung cancer during the peri-pandemic period in northern Poland.

## Materials and methods

### Data collection

The statistical data used in this retrospective analysis were sourced from three major healthcare centers in northern Poland that conduct routine diagnostic procedures for lung cancer detection: the Warmińsko-Mazurski Centre of Pulmonary Diseases in Olsztyn, the Kujawsko-Pomorski Pulmonology Centre in Bydgoszcz, and the GUMed University Clinical Centre in Gdansk.

During the COVID-19 pandemic, the centers in Gdansk and Olsztyn operated relatively normally, with restrictions on admissions imposed only temporarily to mitigate SARS-CoV-2 transmission, rather than due to changes in hospital function. Planned admissions required COVID-19 testing, and a positive result sometimes postponed diagnostic procedures for affected patients. Conversely, in Bydgoszcz, only one of the three wards continued lung cancer diagnostic procedures after March 2020, while the others were dedicated to treating COVID-19 patients. Consequently, the number of admissions dropped by nearly 50% (in 2019–11,067; 2020–6,704; 2021–6,416 new admissions).

This study utilized aggregate data (totaling 15,669 cases) of new, histopathologically confirmed lung cancer cases diagnosed between January 1, 2016, and December 31, 2022, across the three aforementioned healthcare centers. The study was conducted only on numerical data—the number of hospitalizations, without the use or processing of any personal data. No informed consent was obtained. Data for research purposes were accessed from November to December 2023. The study was granted, written, permit no. KB 355/2020 by the Bioethics Committee at Collegium Medicum in Bydgoszcz on March 23, 2020.

### Statistical analysis

Statistical analysis covered patients receiving health services due to cancer from 2016 to 20122 for 3 cities in northern Poland. To determine whether a trend exists in the time series data (2016–2022) for each center in Northern Poland, we used a Mann-Kendall Trend Test at a significance level of p = 0.05. According to the statistical modeling and testing principles in cancer research highlighted by Xu et al., 2021, Relative Risks (RR) with 95% Confidence Intervals (CI) for cancer events were calculated individually for each center (city) and year relative to the COVID-19 period (2020), which was considered as a reference [9].

The study evaluated the number of diagnosed lung cancers and assessed their ratio relative to the population size of the respective city.

The phenomenon of "COVID-19 Debt" in medical practice has been described as the underestimation of lung cancer cases, calculated in relation to the expected trend line for a given center, resulting from limitations in cancer diagnostics during the COVID-19 pandemic.

## Results

Based on the available data from the three healthcare centers in northern Poland that routinely perform lung cancer diagnostic procedures, an analysis was conducted on the lung cancer detection rate from January 1, 2016, to December 31, 2022.

**Fig 1:**
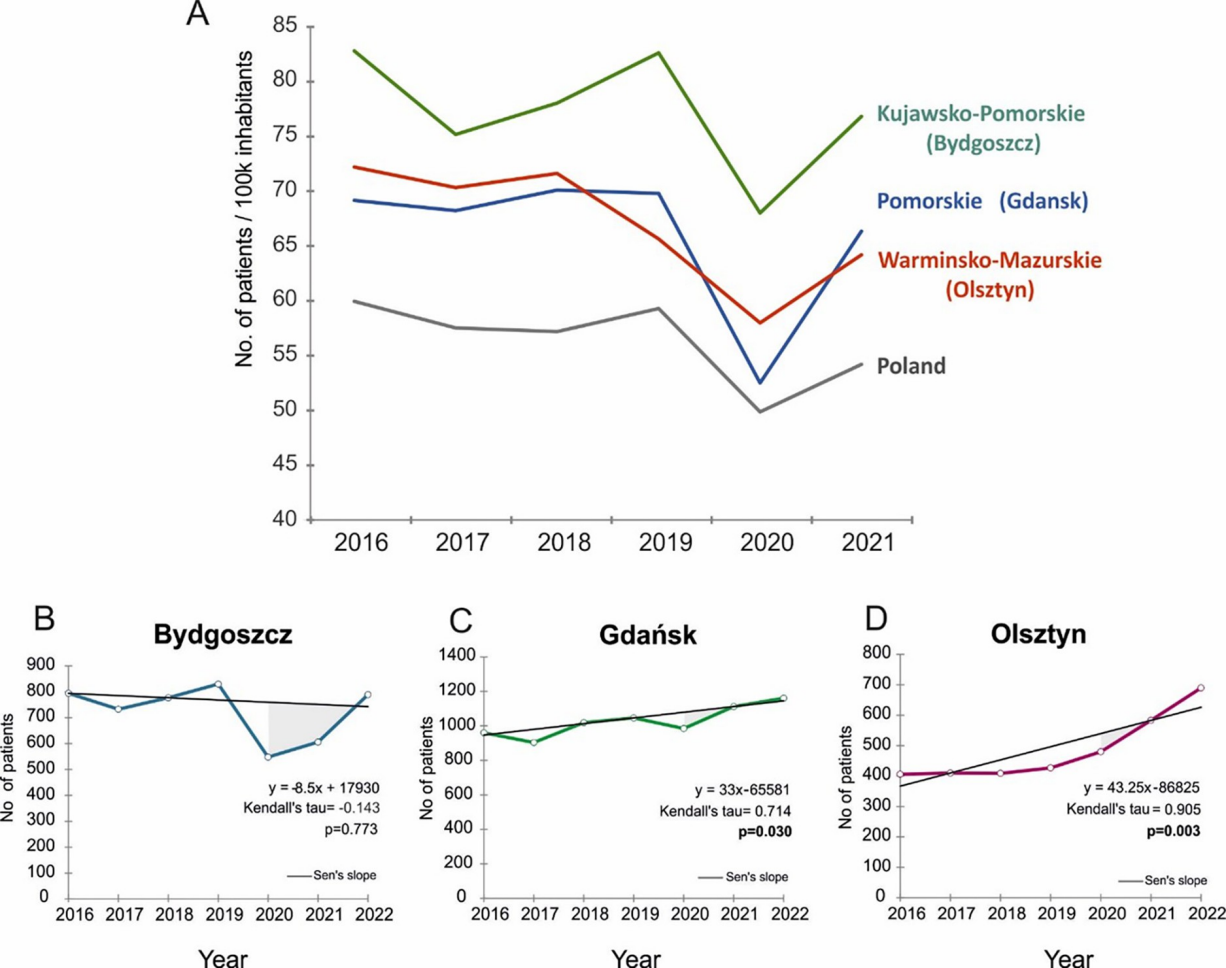
**A—**Curves representing the number of lung cancer diagnoses per 100k inhabitants in consecutive years 2016–2021 derived from the Polish national cancer registry data (2021-last available). B, C, D—Confirmed lung cancer diagnoses in successive years for each of the three centres.

In Fig 1, the quantities of confirmed lung cancer diagnoses in successive years are depicted for each of the three centres based on our data, along with curves representing the number of lung cancer diagnoses derived from the national cancer registry data.

An analysis of the trend in lung cancer detectability reveals intriguing observations (Fig 1), particularly when examined individually for each center (Fig 1B–1D). In all components of Fig 1, particularly in part A relating to data from the national cancer registry, a profound dip is visible in 2020, the year significantly affected by the COVID-19 pandemic. In 2020, the index of patients with lung cancer per 100k inhabitants for Poland dropped by 15% achieving the value of 49.8.

Table 1 displays the number of cases of new diagnosed lung cancers in 3 cities in Poland (Bydgoszcz, Gdansk, and Olsztyn) over the years. In 2020, there was a significant reduction in the number of newly diagnosed lung cancers; in Bydgoszcz, compared to the previous year, the number of diagnoses decreased by 34%, also in Gdańsk, the number of new diagnoses also decreased by almost 6%. The situation is slightly different in Olsztyn, as the number of cancer diagnoses is increasing in 2020, but not as quickly as in subsequent years.

**Table 1:** Confirmed lung cancer diagnoses in successive years for each of the three centres, "COVID-19 debt" (calculated based on Fig 1) marked in bold.

	Number of cases				COVID-19 debt			
City	Bydgoszcz (330 038*)	Gdańsk (486 345)	Olsztyn (168 212)	Sum (984 595)	Bydgoszcz	Gdansk	Olsztyn	Sum
Year								
2016	794	960	406	2160	0.00	-13.00	-39.25	-52.25
2017	733	904	410	2047	52.50	76.00	0.00	128.50
2018	777	1019	409	2205	0.00	-6.00	44.25	38.25
2019	830	1046	427	2303	-61.50	0.00	69.50	8.00
2020	548	985	480	2013	**212.00**	**94.00**	**59.75**	**365.75**
2021	606	1112	583	2301	**145.50**	**0.00**	**0.00**	**145.50**
2022	789	1161	690	2640	**-46.00**	**-16.00**	**-63.75**	**-125.75**

Number of inhabitants [10].

The total number of cases for each year is summarized in the ’Sum’ column, reflecting the cumulative figures across all three cities.

The COVID-19 debt in 2020 is visible in all three centers, and interestingly, in 2022, the opposite effect is already noticeable. This is probably a rebound effect resulting from the Covid-19 debt incurred in 2020—an increase in the number of lung cancer diagnoses in the following years.

Our data also allow us to calculate the relative risk, using the data from 2020 as a control group.

Based on the statistical analysis, a statistically significant trend was determined showing a growth in the incidence of lung cancer in 2021 and 2022 in both Gdansk and Olsztyn centres. No such trend was demonstrated for the Bydgoszcz centre, where, however, there was a significant decrease in the number of diagnosed lung cancer cases in 2020, followed by an increase in the subsequent years, 2021 and 2022. There was a decline in the number of positive diagnoses in 2020 across all three centers, but statistical significance was only observed in Olsztyn (p = 0.003) and Gdansk (p = 0.030).

## Discussion

The results of our study indicate a lower number of positive lung cancer diagnoses in the year 2020, during the COVID-19 pandemic, followed by a considerable increase in subsequent years (Fig 1). The data presented in this paper vary across each of the three centers, which is undoubtedly linked to how hospitals and hospital wards functioned during the pandemic, including the transformation of entire wards and hospitals to treat COVID-19 patients (Table 1).

In 2020, as a result of the COVID-19 pandemic, there was a collapse of the healthcare system. The decrease in the number of diagnosed cases of cancer during this period, referred to as the COVID debt, is noticeable in all three cities, as shown in Table 1. Based on these data, the relative risk was calculated in Table 2, assuming 2020 as the reference year. Our results are unequivocal—in 2022, there is an increase in the number of lung cancer diagnoses, which is a result of the rebound effect of the COVID debt. This rebound effect may be due to disruptions in the health system or an increase in the number of radiological tests performed, but this is only a hypothesis. Additionally, to confirm the data, Fig 1 depicts the trend line for the entire Poland (utilizing available data from the National Cancer Registry), which corroborates our findings [12]. Since collecting data from the country or the world takes time, this study is the first endeavor to evaluate and present the phenomenon of the COVID debt. Undoubtedly, it will also be analyzed in the context of other chronic diseases. In the Warmińsko-Mazurski Centre for Pulmonary Diseases in Olsztyn and the GUMed University Clinical Centre in Gdansk, separate wards were designated for COVID-19 patients, which did not significantly impact the wards conducting diagnostic procedures for suspected lung cancer. However, the Kujawsko-Pomorski Pulmonology Centre in Bydgoszcz operated differently, with nearly the entire center transformed into a ’COVID-19’ hospital, resulting in a significant decrease in diagnosed lung cancer cases, with only one department functioning in normal admission mode.

**Table 2:** Changes in relative risks (RR) of lung cancer in populations of 3 cities (Bydgoszcz, Gdansk and Olsztyn) in Poland in the period of 2016–2022 (exposed group) in comparison to 2020 during COVID-19 (control group). The RR and 95% confidence interval (CI) are calculated according to Altman 1991 [11]. Statistical significance at p<0.05 is depicted in bold.

Year/City	Relative risk				
Bydgoszcz	RR	-95%CI	+95%CI	z	p
2016	1.45	1.30	1.62	6.68	**<0.001**
2017	1.34	1.20	1.49	5.16	**<0.001**
2018	1.42	1.27	1.58	6.27	**<0.001**
2019	1.51	1.36	1.69	7.55	**<0.001**
**2020**	1.00				
2021	1.11	0.99	1.24	1.71	0.088
2022	1.44	1.29	1.61	6.56	**<0.001**
**Gdańsk**					
2016	0.97	0.89	1.07	-0.57	0.570
2017	0.92	0.84	1.00	-1.86	0.063
2018	1.03	0.95	1.13	0.76	0.447
2019	1.06	0.97	1.16	1.35	0.176
**2020**	1.00				
2021	1.13	1.04	1.23	2.77	**0.006**
2022	1.18	1.08	1.28	3.80	**0.000**
**Olsztyn**					
2016	0.85	0.74	0.97	-2.49	**0.013**
2017	0.85	0.75	0.97	-2.35	**0.019**
2018	0.85	0.75	0.97	-2.38	**0.017**
2019	0.89	0.78	1.01	-1.76	0.078
**2020**	1.00				
2021	1.21	1.08	1.37	3.16	**0.002**
2022	1.44	1.28	1.62	6.12	**<0.001**
**Sum**					
2016	1.07	1.01	1.14	2.28	**0.023**
2017	1.02	0.96	1.08	0.54	0.591
2018	1.10	1.03	1.16	2.96	**0.003**
2019	1.14	1.08	1.21	4.42	**<0.001**
**2020**	1.00				
2021	1.14	1.08	1.21	4.39	**<0.001**
2022	1.31	1.24	1.39	9.17	**<0.001**

The decrease in diagnosed lung cancer cases at the Gdansk center in 2020 (Fig 1C) and the lack of a significant increase in new cases diagnosed at the Olsztyn center (Fig 1D) could be attributed to several factors related to the COVID-19 pandemic. These factors include the discontinuation of inpatient medical consultations, delays in diagnostic testing, and patients avoiding healthcare visits [13, 14]. Importantly, the reduction in medical consultations during the pandemic was consistent across different patient demographics, including age, sex, place of residence, and medical specialty of doctors [15].

Piwkowski et al. demonstrated a clear decrease in referrals for surgical treatment of lung cancer during the pandemic, leading to delayed diagnostic procedures and increased cancer mortality (Piwkowski et al., 2022). Additionally, referrals for other diagnostic procedures such as laboratory tests and imaging tests also declined. Van Haren et al., in a study published in the Journal of the American College of Surgeons (JACS), reported significant challenges in lung cancer screening due to COVID-19, resulting in reduced new patient screenings and a higher percentage of diagnosed malignant tumors after resuming screening [16]. The initial decrease in the diagnosis of high-risk cancer tumors was notable, reaching 21% (from 29% to 8%) during the pandemic [16].

The fear of contracting COVID-19 when visiting healthcare centers contributed to patients’ reluctance to seek medical care, potentially leading to delays in cancer diagnosis and treatment. Montalto et al. in a study conducted on 2376 patients from 27 Italian hospitals demonstrated that over half of these patients were concerned about COVID-19 virus transmission [17]. According to a report by Deml et al. in April 2022, many patients still perceived hospitals as risky environments with a higher risk of COVID-19 infection [18]. The fear of hospitalization particularly affected better-educated young adults and older individuals with multiple chronic conditions [19]. The findings of our study highlight the complex interplay between the COVID-19 pandemic and the diagnosis of lung cancer. The initial decrease in diagnosed cases during the pandemic reflects the profound disruptions in healthcare delivery, including reduced medical consultations, delays in diagnostic procedures, and patient avoidance of healthcare settings. These challenges likely contributed to missed or delayed diagnoses of lung cancer, potentially impacting patient outcomes.

On the other hand, the COVID-19 pandemic has contributed to a subsequent increase in the number of diagnosed cancer cases in the post-pandemic years. This trend was observed across all three centers, calculated per 100,000 residents (Fig 1A), particularly evident in Olsztyn and Gdansk (Tables 1 and 2). Concerning the Bydgoszcz center (Fig 1B), it is crucial to maintain ongoing surveillance of this trend, as it might exhibit a delayed response compared to Olsztyn and Gdansk, where the impact of the COVID-19 pandemic on healthcare centers was less severe, as evidenced by our results (Tables 1 and 2).

The increase in newly diagnosed cases of lung cancer likely resulted from improved access to doctors and pulmonologists during the post-pandemic years, allowing for the detection of illnesses that developed during the COVID-19 pandemic. However, there is a question of whether the COVID-19 pandemic may have had a positive effect by enhancing oncological vigilance. COVID-19 predominantly affects the respiratory system, and severe cases leading to acute respiratory distress syndrome (ARDS) in up to 16% of patients increased referrals for imaging tests [20]. Additionally, patients with persistent long-term COVID-19 symptoms or imaging findings after COVID-19 infection were referred to specialist pulmonology clinics, further increasing oncological vigilance.

The increase in newly diagnosed lung cancer cases can be attributed to the "COVID-19 debt" or seen as a phenomenon reflecting heightened medical attention post-pandemic. Here, the term "COVID-19 debt" signifies a delay or gap in the statistical understanding and public health reporting of lung cancer incidences during and potentially following the COVID-19 years. This emphasizes the necessity of continuous surveillance and proactive healthcare measures to address delayed cancer diagnoses during the pandemic.

Moving forward, healthcare systems must prioritize strategies aimed at mitigating the long-term impact of the pandemic on cancer care. This includes adopting innovative approaches to healthcare delivery, enhancing screening programs, and promoting patient education to facilitate timely diagnosis and treatment of lung cancer and other malignancies. Collaborative efforts among healthcare providers, policymakers, and community stakeholders are crucial to navigate the challenges posed by the pandemic and ensure optimal cancer care outcomes in the future.

## Limitations

The impact of the COVID-19 pandemic on the operational strategies of internal medicine and pulmonology wards was profound and widespread globally, resulting in substantial disruptions to diagnostic processes and patient care volumes. Although our data originate from specific centers in northern Poland, they are indicative of broader trends experienced across healthcare systems during the pandemic. These limitations highlight the need for cautious interpretation of our findings within the context of global healthcare challenges posed by the pandemic. In the current healthcare framework in Poland, patients possess the autonomy to select their treatment facility without regard to their residential location. Naturally, regional centers garner greater preference, particularly in densely populated urban areas like Gdansk, where such facilities are more abundant. Consequently, our dataset is not conducive to presenting incidence figures in a conventional manner. Nonetheless, through preliminary computations, these data offer an approximation of regional incidence rates. We abstain from presenting this information, as we believe it would be erroneous to do so. Instead, we rely on data sourced from the national cancer registry, current as of 2021. We include city population size to provide readers with a frame of reference.

### Summary

In summary, the COVID-19 pandemic initially resulted in decreased lung cancer detection rates, influenced by operational changes within healthcare centers. However, there was subsequently an increase in lung cancer diagnoses post-pandemic, possibly due to heightened societal vigilance and increased diagnostic efforts. This increase highlights the importance of implementing post-pandemic healthcare strategies focused on enhancing cancer detection and improving patient outcomes.
